# Clinical feasibility of (neo)adjuvant taxane-based chemotherapy in older patients: analysis of >4,500 patients from four German randomized breast cancer trials

**DOI:** 10.1186/bcr2144

**Published:** 2008-09-16

**Authors:** Sibylle Loibl, Gunter von Minckwitz, Nadia Harbeck, Wolfgang Janni, Dirk Elling, Manfred Kaufmann, Holm Eggemann, Valentina Nekljudova, Harald Sommer, Marion Kiechle, Sherko Kümmel

**Affiliations:** 1 German Breast Group, Schleussnerstrasse 42, Neu-Isenburg 63263, Germany; 2 Department of Obstetrics and Gynecology, J.W. Goethe-University, Theodor-Stern Kai 7, Frankfurt 60590, Germany; 3 Department of Obstetrics and Gynecology, Technical University of Munich, Ismaninger Strasse 22, Munich 81675, Germany; 4 Department of Obstetrics and Gynecology, Ludwig-Maximilians University, Maistrasse 11, Munich 8337, Germany; 5 Department of Obstetrics and Gynecology, Otto-von-Guericke University, Gerhart-Hauptmann Strasse 35, 39108 Magdeburg, Germany; 6 Department of Obstetrics and Gynecology, University Hospital Essen, Hufelandstrasse 55, Essen 45122, Germany

## Abstract

**Introduction:**

Despite the fact that people older than 65 years of age have the highest incidence of developing breast cancer, these patients are excluded from clinical trials in most cases. Furthermore, most physicians tend towards therapy regimens without the use of dose-dense, highly active taxane-based treatments because of a lack of data regarding toxicities of these compounds in older patients.

**Methods:**

Pooled side-effect data were analyzed from four prospective, randomized clinical trials in which patients of different age groups (< 60 years, between 60 and 64 years, and > 64 years) with primary breast cancer received taxane-based chemotherapy.

**Results:**

Dose delays, dose reductions, hospitalization, and therapy discontinuation increased with age. Hematologic toxicities and some nonhematologic toxicities were generally more common in older patients. Leucopenia increased from 55.3% in patients aged < 60 years to 65.5% in patients aged > 64 years (*P *< 0.001), and neutropenia increased from 46.9% to 57.4% (*P *< 0.001). There was no difference, however, in clinically more relevant febrile neutropenia between the different age groups. Thrombopenia shows a similar age-dependent increase, whereas there is no difference between the age groups concerning anemia. Hot flushes and elevated liver enzymes decreased with increasing age.

**Conclusions:**

The present pooled analysis of a substantial cohort of older primary breast cancer patients demonstrates that taxane-containing (neo)adjuvant chemotherapy is feasible in older patients and that toxicity can be reduced by sequential therapy regimens.

## Introduction

The incidence of breast cancer in women aged 65 years and older is the highest in all age groups. These older patients are generally underrepresented or even excluded from clinical trials, however, leading to a gap in data about the compliance, safety, and efficacy of highly active taxane-based treatments in older patients. From several clinical trials, there is a growing awareness that older primary breast cancer patients achieve significant survival benefits with (neo)adjuvant chemotherapy regimens. Regarding the steady increase of life expectancy today, this should be considered in treatment of older patients in clinical routine.

There is evidence in breast cancer that the use of taxanes in adjuvant chemotherapy yields a survival benefit – especially from the PACS 01 study, demonstrating a particular benefit in women aged 50 years or older [1]. There are relatively few data, however, on the use of these agents in older patients [2]. Owing to concerns about tolerability, there remains a tendency in clinical routine to use less dose-intensive chemotherapy in older patients with the avoidance of those agents perceived to be more toxic. We therefore combined data of different adjuvant and neoadjuvant trials with taxane-containing regimens. Here we present the results of the first systematic pooled analysis of tolerability data for taxane-based regimens in older primary breast cancer patients.

## Materials and methods

**Figure 1:**
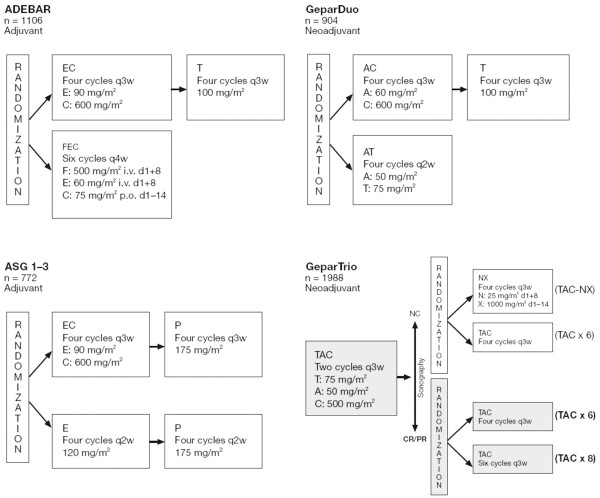
Study design of the four trials. CR, complete response; PR, partial response; NC, no clinical response. EC-T, epirubicin, cyclophosphamide followed by docetaxel; FEC, 5-fluorouracil, epirubicin, cyclophosphamide; EC-P, epirubicin, cyclophosphamide followed by paclitaxel; AC-T, doxorubicin, cyclophosphamide followed by docetaxel; E-P, epirubicin followed by paclitaxel; AT, doxorubicin, docetaxel; TAC, docetaxel, doxorubicin, cyclophosphamide; NX, vinorelbine, capecitabine. q2W, every 2 weeks; q3W, every 3 weeks; q4W, every 4 weeks. i.v., intravenously; p.o., per orally; d, day.

A pooled analysis of four German prospective, randomized clinical trials, conducted during 1999 to 2005, was performed. These trials included primary breast cancer patients receiving taxane-containing neoadjuvant or adjuvant chemotherapy. The meta-database was closed in October 2006; the number of patients in the present analysis may therefore differ from the separate study publications. For every study, however, the number of patients included in the present analysis exceeds 75% of those evaluable. Toxicity data from the studies were analyzed for taxane-containing chemotherapy in older patients (aged > 64 years) compared with toxicity data from patients aged < 60 years and those aged 60 to 64 years treated in the same studies. The treatment regimens of the four trials included are depicted in Figure 1.

In the ADEBAR trial (NCT00047099), patients (*n* = 1,106/1,502) received four cycles of adjuvant chemotherapy either with epirubicin/cyclophosphamide every 3 weeks followed by four cycles of docetaxel every 3 weeks, or six cycles of 5-fluorouracil, epirubicin on days 1 and 8 and cyclophosphamide on days 1 to 14 every 4 weeks [3].

In the ASG 1–3 trial (NCT00668616), patients (*n* = 772) received four cycles of adjuvant chemotherapy either with epirubicin/cyclophosphamide every 3 weeks then four cycles of paclitaxel every 3 weeks, or with four cycles of epirubicin every 2 weeks and then four cycles of paclitaxel every 2 weeks (unpublished data, Kümmel S. *et al*).

In the GeparDuo trial (NCT00543829), patients (*n* = 902) received four cycles of neoadjuvant chemotherapy with doxorubicin/cyclophosphamide every 3 weeks followed by four cycles of docetaxel every 3 weeks or four cycles of doxorubicin/docetaxel every 2 weeks [4].

In the GeparTrio trial (NCT00544765), patients (*n* = 1,988/2,072) received two cycles of neoadjuvant chemotherapy with docetaxel/doxorubicin/cyclophosphamide (TAC) followed by either four cycles of TAC or six cycles of TAC or four cycles of vinorelbine plus capecitabine every 3 weeks [5,6].

For the purpose of the analysis, chemotherapy regimens were divided into four chemotherapy schedules: combination taxane schedule, TAC 75/50/600 mg/m^2^; sequence schedule, doxorubicin(epirubicin)cyclophosphamide 60(90)/600 mg/m^2 ^followed by docetaxel 100 mg/m^2 ^or paclitaxel 175 mg/m^2^; combination dose-dense schedule, dose-dense doxorubicin/docetaxel 50/75 mg/m^2^; and sequence dose-dense schedule, dose-dense epirubicin/dose-dense paclitaxel 120/175 mg/m^2^.

### Supportive care during the studies

Various strategies for supportive therapy and premedication were used in the studies analyzed. All patients were given prophylactic 5-HT_3 _antagonists, however, and all patients receiving taxane regimens also received dexamethasone. Primary neutropenia prophylaxis was not administered to patients receiving nontaxane chemotherapy and was not mandatory for patients receiving the sequence schedule. All patients receiving combination dose-dense schedules and sequential dose-dense schedules received prophylaxis with filgrastim or lenograstim on days 5 to 10. Among the patients receiving the combination taxane schedule, 16% received no primary prophylaxis with granulocyte-colony stimulating factor, 23% received filgrastim or lenograstim on days 5 to 10, and 61% received pegfilgrastim on day 2 [7]. No patients receiving the sequence schedule, the combination dose-dense schedule or the sequence dose-dense schedule received primary anti-infective prophylaxis – while among those patients who received the combination taxane schedule, 44% received no prophylaxis and 56% received ciprofloxacin on days 5 to 14. In the GeparTrio study, supportive care changed during the study from ciprofloxacin alone in the pilot phase to filgrastim or lenograstim prophylaxis, then to pegfilgrastim and, finally, to pegfilgrastim plus ciprofloxacin [7].

### Data collection and statistical analyses

Data were collected on dose delays/reductions, hospitalizations, treatment discontinuation, deaths, and hematologic and nonhematologic toxicity. For hematologic toxicity, not all records of all cycles in the four studies included the same data on events: febrile neutropenia (FN) data were recorded for patients on the TAC regimen; all other patients were considered to have FN of at least grade 3 in a given chemotherapy cycle if they had grade 3/4 neutropenia, more than grade 1 fever, and no infection. All FN cases reported as serious adverse events with severity grade were also considered. In cycles where at least one of the three parameters (neutropenia, fever, infection) was missing, and FN was not reported in the serious adverse events description, the cycle was considered a missing value for FN.

All statistical analyses were exploratory and no adjustments were made for multiple comparison. Calculations were performed using SPSS 12.0.1 for Windows (SPSS Inc. Chicago, IL, USA). Grading systems for toxicities in different studies were checked for consistency and were converted into NCI-CTCAE 3.0 grades. Pearson's chi-squared test was performed to compare incidences of toxicity endpoints in the three different age groups of patients.

## Results

Across the four studies, 422 patients aged ≥ 65 years (out of 4,227 patients), with a median age of 67 years (range 65 to 80 years), received 1,674 cycles of taxane-containing chemotherapy regimens. Furthermore, 3,160 patients aged < 60 years, with a median age of 47 years (range 23 to 59 years), received 14,146 cycles of taxane-containing chemotherapy regimens. Across the studies, 2,674 cycles were given to patients aged between 60 and 64 years. Demographic and clinical characteristics of the patients who received a taxane-containing chemotherapy and the summary data for all 'older' patients (aged > 64 years and aged 61 to 64 years) and 'younger' patients (aged < 60 years) are presented in Table 1.

**Table 1:** Demographic and clinical characteristics of patients at baseline

Characteristic	Age group			Total
		
	< 60 years	60 to 64 years	> 64 years	
Cases				
Total	3,160 (100)	645 (100)	422 (100)	4,227 (100)
Receptor status (ER-positive and/or PgR-positive versus both negative)				
Positive	1,941 (61.4)	447 (69.3)	278 (65.9)	2,666 (63.1)
Negative	876 (27.7)	144 (22.3)	112 (26.5)	1,132 (28.6)
Unknown	343 (10.9)	54 (8.4)	32 (7.6)	429 (10.1)
Tumor grading				
Grade 1	142 (5.1)	29 (5.0)	22 (5.8)	193 (5.2)
Grade 2	1,534 (54.9)	321 (55.6)	224 (59.4)	2,079 (55.5)
Grade 3	1,117 (40.0)	227 (39.3)	131 (34.7)	1,475 (39.4)
Valid	2,793 (88.4)	577 (89.5)	377 (89.3)	3,747 (88.6)
Missing	367 (11.6)	68 (10.5)	45 (10.7)	480 (11.4)
Histological tumor type				
Ductal invasive	2,405 (76.1)	459 (71.1)	315 (74.6)	3,179 (75.2)
Lobular invasive	477 (15.0)	127 (19.6)	65 (15.4)	669 (15.8)
Other	233 (7.3)	54 (8.3)	35 (8.2)	322 (7.6)
Valid	3,115 (98.6)	640 (99.9)	415 (98.4)	4,170 (98.7)
Missing	45 (1.4)	5 (0.1)	7 (1.6)	57 (1.3)
Surgery type				
Breast-conserving	1935 (61.2)	381 (59.0)	228 (54.0)	2,544 (60.1)
Mastectomy	961 (30.4)	216 (33.4)	161 (38.1)	1,338 (31.6)
Valid	2,896 (91.6)	597 (92.5)	389 (92.1)	3,882 (91.8)
Missing	264 (8.3)	48 (7.4)	33 (7.8)	345 (8.1)
Age median (years)	47	62	67	51
Age range (years)	23 to 59	60 to 64	65 to 80	23 to 80

Data presented as *n *(%). All percentage values are valid. ER, estrogen receptor; PgR, progesterone receptor.

### Dose delays/reductions, hospitalizations, therapy discontinuations, and deaths during the trials

**Figure 2:**
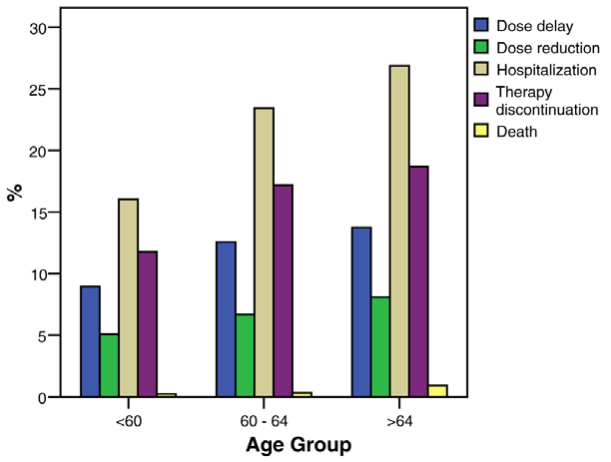
Dose reduction, dose delay, hospitalization, therapy discontinuation, and death per patient, versus age group.

Dose delays (evaluated from first taxane cycle in sequential regimens), reductions, and therapy discontinuation for any reason increased with age (Figure 2).

**Figure 3:**
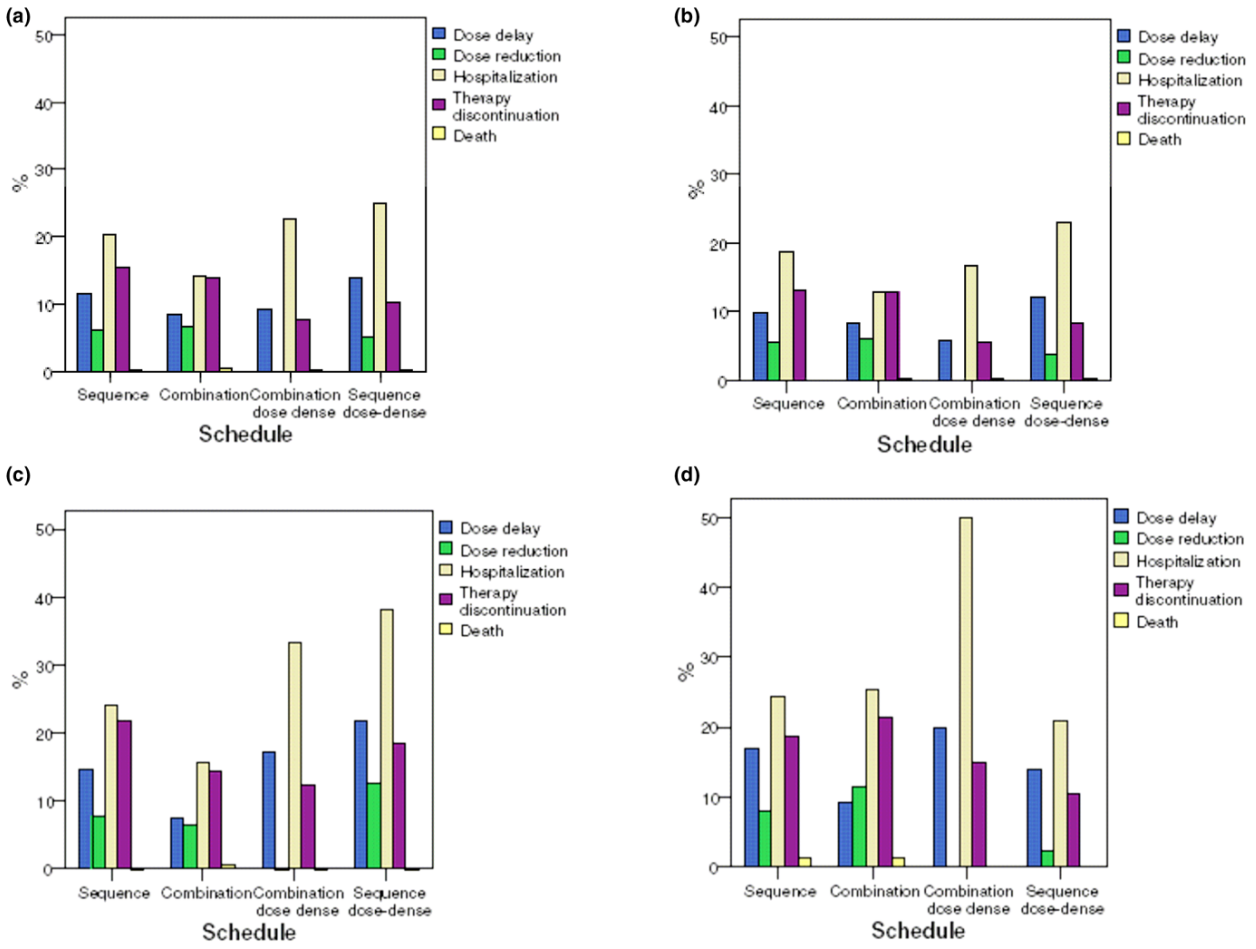
Dose reduction, dose delay, hospitalization, therapy discontinuation, and death versus schedule. **(a) **For all patients. **(b) **For patients younger than 60 years old. **(c) **For patients aged 60 to 64 years. **(d) **For patients older than 64 years of age.

Overall during the taxane cycles, dose delays were reported in 9.0% of patients younger than 60 years versus 12.6% of patients aged between 60 and 64 years and 13.7% of patients aged 65 years and older (*P *= 0.001). Dose reductions were reported in 5.1% of patients aged < 60 years, 6.7% of patients aged 60 to 64 years, and 8.1% of patients aged ≥ 65 years (*P *= 0.019), hospitalization was reported in 16.0%, 23.4%, and 18.1% (*P *< 0.001), treatment discontinuation was reported in 11.8%, 17.2%, and 18.7% (*P *< 0.001), and deaths were reported in 0.2%, 0.3%, and 1.0%, respectively (Figure 3a).

Overall among taxane schedules, the incidences of dose delays (13.8%) and of hospitalization (25%) were markedly higher with the sequence dose-dense schedule (dose-dense epirubicin/dose-dense paclitaxel regimen) versus other taxane schedules. There are, however, age-specific differences. The patient age groups < 60 years and 60 to 64 years performed similarly, whereas patients older than 64 years had the highest incidences of dose delays (20%) and hospitalizations (50%) with the combination dose-dense schedule (dose-dense doxorubicin/docetaxel) (Figure 3b,c,d).

### Hematological toxicity

**Figure 4:**
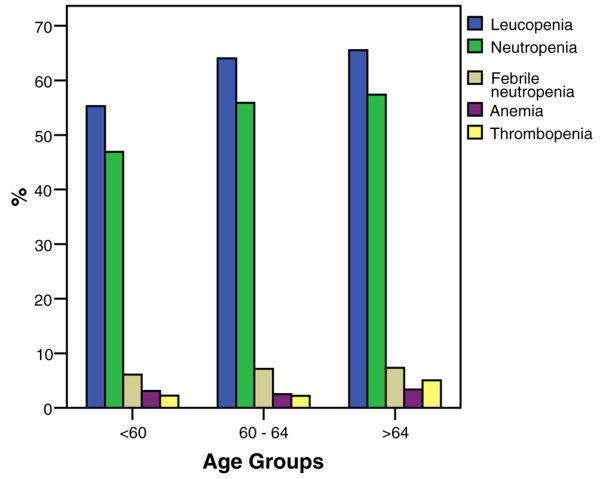
Incidences of grade 3 and grade 4 hematologic adverse events versus age group. Leucopenia: age <60 years, *n* = 1,730; age between 60 and 64 years, *n* = 406; age >64 years, *n* = 272 (*P *< 0.001). Neutropenia: age <60 years, *n* = 1,152; age between 60 and 64 years, *n* = 326; age >64 years, *n* = 225 (*P *< 0.001). Febrile neutropenia: age <60 years, *n* = 181; age between 60 and 64 years, *n* = 43; age >64 years, *n* = 29 (*P *= 0.430). Anemia: age <60 years, *n* = 97; age between 60 and 64 years, *n* = 16; age >64 years, *n* = 14 (*P *= 0.674). Thrombopenia: age <60 years, *n* = 70; age between 60 and 64 years, *n* = 14; age >64 years, *n* = 21 (*P *= 0.002).

In older patients versus younger patients, the per-patient incidences of grade 3 to grade 4 hematologic adverse events generally increased with age (Figure 4). Leucopenia increased from 55.3% in patients < 60 years old to 65.5% in patients > 64 years old (*P *< 0.001), and neutropenia increased from 46.9% to 57.4% (*P *< 0.001). There was no difference, however, in FN between the different age groups.

For taxane therapy, the incidences of grade 3 to grade 4 leucopenia were all statistically significantly different per patient between the age groups and for docetaxel-treated patients (*P *< 0.001), whereas for paclitaxel-treated patients an age difference was only seen for leucopenia (Table 2). Overall there was no age difference in developing FN. In patients younger than 60 years of age FN was only significantly more common in the docetaxel-containing regimen, whereas there was no difference between the docetaxel and paclitaxel regimen in patients older than 60 years. Anemia and thrombopenia were significantly more common in the paclitaxel-containing regimen but only in the age group below 60 years of age. The rate of thrombopenia increased with age, whereas there is no age difference with anemia.

**Table 2:** Incidence of grade 3 to grade 4 hematologic toxicity, per age interval and per taxane

	Docetaxel	Paclitaxel	Total	*P *value, docetaxel versus paclitaxel
Leukopenia				
Patients aged < 60 years	1,534 (59.6)	196 (35.3)	1,730 (55.3)	<0.001
Patients aged 60 to 64 years	351 (68.3)	55 (45.8)	406 (64.0)	<0.001
Patients aged > 64 years	234 (69.2)	38 (49.4)	272 (65.5)	0.001
*P *value	<0.001	0.011	<0.001	
Neutropenia				
Patients aged < 60 years	926 (47.4)	226 (44.8)	1,152 (46.9)	0.321
Patients aged 60 to 64 years	273 (57.6)	53 (48.6)	326 (55.9)	0.111
Patients aged > 64 years	190 (59.2)	35 (49.3)	225 (57.4)	0.164
*P *value	<0.001	0.643	<0.001	
Febrile neutropenia				
Patients aged <60 years	179 (7.2)	2 (0.4)	181 (6.1)	<0.001
Patients aged 60 to 64 years	40 (8.1)	3 (2.8)	43 (7.2)	0.085
Patients aged > 64 years	28 (8.7)	1 (1.4)	29 (7.4)	0.061
*P *value	0.560	0.049	0.430	
Anemia				
Patients aged < 60 years	57 (2.2)	40 (7.2)	97 (3.1)	<0.001
Patients aged 60 to 64 years	12 (2.3)	4 (3.3)	16 (2.5)	0.756
Patients aged > 64 years	12 (3.6)	2 (2.6)	14 (3.4)	0.946
*P *value	0.315	0.110	0.674	
Thrombopenia				
Patients aged < 60 years	44 (1.7)	26 (4.7)	70 (2.3)	<0.001
Patients aged 60 to 64 years	13 (2.5)	1 (0.8)	14 (2.2)	0.432
Patients aged > 64 years	21 (6.2)	0 (0.0)	21 (5.1)	0.050
*P *value	<0.001	0.025	0002	

Data presented as *n *(%). All percentage values are valid. *P *values all for incidence of difference between age groups.

Among taxane schedules, the taxane combination (TAC) was overall associated with the highest incidence of hematologic toxicity, and the dose-dense paclitaxel part of the sequence dose-dense schedule with the lowest incidence (Table 3). For the TAC regimen, grade 3 to grade 4 leucopenia was reported in 57.3% of patients < 60 years old, 65.3% for the patient group between 60 and 64 years old, and 64.9% for the patients older than 64 years. Similar results are presented for grade 3 to grade 4 neutropenia in 37.5%, and 56.7%, and 55.2% and for FN in 9.3%, 11.3%, and 14.3% for the different age groups, respectively. Thrombopenia shows a similar age-dependent increase, whereas there is no difference between the age groups concerning anemia.

**Table 3:** Incidence of leukopenia and neutropenia per schedule according to different age intervals

	Combination (TAC) schedule (*n* = 450)	Sequence schedule (*n* = 360)		Combination dose-dense (ATdd) schedule (*n* = 121)	Sequence dose-dense schedule (*n* = 86)	
				
		Nontaxane	Taxane		Nontaxane (dose-dense epirubicin)	Taxane (dose-dense paclitaxel)
Leukopenia						
Patients aged < 60 years	878 (57.3)	423 (43.4)	419 (47.3)	164 (50.2)	82 (29.2)	7 (2.6)
Patients aged 60 to 64 years	175 (65.3)	124 (53.9)	107 (51.0)	49 (61.3)	20 (36.4)	0 (0.0)
Patients aged > 64 years	111 (64.9)	99 (60.7)	71 (49.0)	25 (62.5)	17 (42.5)	1 (2.9)
Neutropenia						
Patients aged < 60 years	371 (37.5)	455 (52.8)	346 (45.3)	136 (43.5)	74 (29.7)	20 (8.7)
Patients aged 60 to 64 years	139 (56.7)	106 (52.0)	79 (44.1)	37 (47.4)	18 (36.7)	2 (5.0)
Patients aged > 64 years	91 (55.2)	97 (65.5)	61 (48.4)	17 (43.6)	11 (33.3)	1 (3.2)
Febrile neutropenia						
Patients aged < 60 years	140 (9.3)	9 (1.0)	19 (2.5)	14 (4.5)	0 (0.0)	0 (0.0)
Patients aged 60 to 64 years	30 (11.3)	1 (0.5)	5 (2.8)	4 (5.1)	3 (6.4)	0 (0.0)
Patients aged > 64 years	24 (14.3)	2 (1.4)	1 (0.8)	2 (5.1)	0 (0.0)	0 (0.0)

Data presented as *n *(%). All percentage values are valid. TAC, docetaxel/doxorubicin/cyclophosphamide; ATdd, doxorubicin/docetaxel dose-dense.

For the dose-dense doxorubicin/docetaxel regimen, grade 3 to grade 4 leucopenia was reported in 50.2%, 61.3%, and 62.5% of patients in the age groups < 60 years, 60 to 64 years and > 64 years. There was no difference between the age groups for neutropenia, FN, and anemia and thrombopenia grade 3 to grade 4.

In contrast to the combination schedules, the highest incidence of grade 3 to grade 4 toxicity in the sequence schedules occurred for neutropenia, which was reported in 65.5% of patients older than 64 years when treated with the nontaxane-containing regimen (epirubicin/cyclophosphamide or doxorubicin/cyclophosphamide). The sequential paclitaxel regimen was also comparatively well tolerated – especially, the sequential dose-dense paclitaxel schedule had significantly fewer toxicity cases compared with the epirubicin dose-dense schedule, with a peak incidence of 8.7% for grade 3 to grade 4 neutropenia per patient (with no cases of FN reported) in the age group younger than 60 years.

### Nonhematologic toxicity

Not all of the nonhematologic toxicity data were consistently recorded for all chemotherapy regimens and cycles in the trials. The nonhematologic toxicity associated with taxane chemotherapy is summarized in Table 4.

**Table 4:** Nonhematologic toxicity per schedule in patients aged ≥ 60 years, n (%)

	Docetaxel	Paclitaxel	Total
Fatigue grade 1 to grade 4			
Patients aged <60 years	1,922 (88.4)	-	1,922 (88.4)
Patients aged 60 to 64 years	362 (87.0)	-	362 (87.0)
Patients aged >64 years	235 (90.4)	-	235 (90.4)
*P *value	0.414	-	0.414
Fatigue grade 3 to grade 4			
Patients aged <60 years	315 (14.5)	-	315 (14.5)
Patients aged 60 to 64 years	81 (19.5)	-	81 (19.5)
Patients aged >64 years	61 (23.5)	-	61 (23.5)
*P *value	<0.001	-	<0.001
Loss of appetite grade 1 to grade 3			
Patients aged <60 years	441 (67.0)	-	441 (67.0)
Patients aged 60 to 64 years	110 (73.8)	-	110 (73.8)
Patients aged >64 years	76 (84.4)	-	76 (84.4)
*P *value	0.002	-	0.002
Loss of appetite grade 3			
Patients aged <60 years	76 (11.6)	-	76 (11.6)
Patients aged 60 to 64 years	33 (22.1)	-	33 (22.1)
Patients aged >64 years	18 (20.0)	-	18 (20.0)
*P *value	0.001	-	0.001
Nausea/vomiting grade 1 to grade 4			
Patients aged <60 years	1,980 (77.5)	473 (84.6)	2,453 (78.8)
Patients aged 60 to 64 years	410 (79.3)	101 (85.6)	511 (80.5)
Patients aged >64 years	290 (85.5)	62 (78.5)	352 (84.2)
*P *value	0.003	0.337	0.028
Nausea/vomiting grade 3 to grade 4			
Patients aged <60 years	206 (8.1)	32 (5.7)	238 (7.6)
Patients aged 60 to 64 years	47 (9.1)	2 (1.7)	49 (7.7)
Patients aged >64 years	34 (10.0)	0 (0.0)	39 (9.3)
*P *value	0.392	0.175	0.479
Mucositis grade 1 to grade 4			
Patients aged <60 years	1,736 (68.0)	299 (53.5)	2,035 (65.4)
Patients aged 60 to 64 years	360 (69.9)	77 (65.3)	437 (69.0)
Patients aged >64 years	231 (68.5)	42 (53.2)	273 (65.6)
*P *value	0.688	0.060	0.205
Mucositis grade 3 to grade 4			
Patients aged <60 years	115 (4.5)	9 (1.6)	124 (4.0)
Patients aged 60 to 64 years	26 (5.0)	7 (5.9)	33 (5.2)
Patients aged >64 years	25 (7.4)	5 (6.3)	30 (7.2)
*P *value	0.064	0.004	0.007
Sensory neuropathy grade 1 to grade 4			
Patients aged <60 years	39 (1.5)	15 (2.7)	54 (1.7)
Patients aged 60 to 64 years	16 (3.1)	2 (1.7)	18 (2.8)
Patients aged >64 years	6 (1.8)	3 (3.8)	9 (2.2)
*P *value	0.729	0.803	0.647
Sensory neuropathy grade 3 to grade 4			
Patients aged <60 years	39 (1.5)	15 (2.7)	54 (1.7)
Patients aged 60 to 64 years	16 (3.1)	2 (1.7)	18 (2.8)
Patients aged >64 years	6 (1.8)	3 (3.8)	9 (2.2)
*P *value	0.050	0.662	0.177
Conjunctivitis grade 1 to grade 3			
Patients aged <60 years	1,002 (46.1)	-	1,002 (46.1)
Patients aged 60 to 64 years	191 (45.9)	-	191 (45.9)
Patients aged >64 years	119 (45.9)	-	119 (45.9)
*P *value	0.996	-	0.996
Conjunctivitis grade 3			
Patients aged <60 years	25 (1.2)	-	25 (1.2)
Patients aged 60 to 64 years	7 (1.7)	-	7 (1.7)
Patients aged >64 years	4 (1.5)	-	4 (1.5)
*P *value	0.615	-	0.615
Nail changes grade 1 to grade 3			
Patients aged <60 years	849 (39.1)	-	849 (39.1)
Patients aged 60 to 64 years	171 (41.3)	-	171 (41.3)
Patients aged >64 years	100 (38.8)	-	100 (38.8)
*P *value	0.680	-	0.680
Nail changes grade 3			
Patients aged <60 years	22 (1.0)	-	22 (1.0)
Patients aged 60 to 64 years	6 (1.4)	-	6 (1.4)
Patients aged >64 years	1 (0.4)	-	1 (0.4)
*P *value	0.411	-	0.411
Skin changes grade 1 to grade 4			
Patients aged <60 years	1,126 (44.1)	202 (36.1)	1,328 (42.7)
Patients aged 60 to 64 years	221 (42.9)	51 (43.2)	272 (43.0)
Patients aged >64 years	137 (40.8)	25 (31.6)	162 (39.0)
*P *value	0.479	0.212	0.348
Skin changes grade 3 to grade 4			
Patients aged <60 years	43 (1.7)	4 (0.7)	47 (1.5)
Patients aged 60 to 64 years	19 (3.7)	2 (1.7)	21 (3.3)
Patients aged >64 years	12 (3.6)	0 (0.0)	12 (2.9)
*P *value	0.003	0.388	0.003
Diarrhea grade 1 to grade 4			
Patients aged <60 years	1,037 (40.6)	137 (24.5)	1,174 (37.7)
Patients aged 60 to 64 years	219 (42.2)	30 (25.4)	249 (39.3)
Patients aged >64 years	171 (50.6)	13 (16.5)	184 (44.1)
*P *value	0.002	0.263	0.039
Diarrhea grades 3 to grade 4			
Patients aged <60 years	99 (3.9)	7 (1.3)	106 (3.4)
Patients aged 60 to 64 years	24 (4.7)	0 (0.0)	24 (3.8)
Patients aged >64 years	17 (5.0)	0 (0.0)	17 (4.1)
*P *value	0.482	0.288	0.731
Fluid retention grade 1 to grade 4			
Patients aged <60 years	954 (37.4)	-	954 (37.4)
Patients aged 60 to 64 years	188 (36.5)	-	188 (36.5)
Patients aged >64 years	128 (38.0)	-	128 (38.0)
*P *value	0.898	-	0.898
Fluid retention grade 3 to grade 4			
Patients aged <60 years	42 (1.6)	-	42 (1.6)
Patients aged 60 to 64 years	7 (1.4)	-	7 (1.4)
Patients aged >64 years	4 (1.2)	-	4 (1.2)
*P *value	0.754	-	0.754
Infection with neutropenia grade 1 to grade 4			
Patients aged <60 years	174 (16.7)	36 (6.4)	210 (13.1)
Patients aged 60 to 64 years	48 (19.4)	15 (12.5)	63 (17.2)
Patients aged >64 years	35 (21.0)	3 (3.8)	38 (15.4)
*P *value	0.305	0.029	0.104
Infection with neutropenia grade 3 to grade 4			
Patients aged <60 years	21 (2.0)	4 (0.7)	25 (1.6)
Patients aged 60 to 64 years	9 (3.6)	3 (2.5)	12 (3.3)
Patients aged >64 years	8 (4.8)	2 (2.5)	10 (4.1)
*P *value	0.062	0.129	0.010
Creatinine grade 1 to grade 4			
Patients aged <60 years	73 (3.4)	7 (1.3)	80 (3.0)
Patients aged 60 to 64 years	26 (6.3)	5 (4.2)	31 (5.8)
Patients aged >64 years	20 (7.8)	5 (7.0)	25 (7.6)
*P *value	<0.001	0.003	<0.001
Hot flushes grade 1 to grade 3			
Patients aged <60 years	425 (64.6)	-	425 (64.6)
Patients aged 60 to 64 years	88 (59.1)	-	88 (59.1)
Patients aged >64 years	46 (51.1)	-	46 (51.1)
*P *value	0.031	-	0.031
Hot flushes grade 3			
Patients aged <60 years	66 (10.0)	-	66 (10.0)
Patients aged 60 to 64 years	15 (10.1)	-	15 (10.1)
Patients aged >64 years	2 (2.2)	-	2 (2.2)
*P *value	0.053	-	0.053
Liver enzymes grade 1 to grade 4			
Patients aged <60 years	1,198 (47.2)	447 (82.8)	1,645 (53.5)
Patients aged 60 to 64 years	193 (42.0)	95 (84.1)	288 (50.3)
Patients aged >64 years	111 (35.7)	53 (74.6)	164 (42.9)
*P *value	<0.001	0.206	<0.001
Liver enzymes grade 3 to grade 4			
Patients aged <60 years	48(1.9)	65 (12.0)	113 (3.7)
Patients aged 60 to 64 years	12 (2.6)	10 (8.8)	22 (3.8)
Patients aged >64 years	7 (2.3)	0 (0.0)	7 (1.8)
*P *value	0.574	0.006	0.166

Data presented as *n *(%). All percentage values are valid.

In older patients versus younger patients there were significantly higher incidences (*P *< 0.05) of grade 3 to grade 4 fatigue (14.5% in patients aged < 60 years versus 19.5% in patients aged 60 to 64 years versus 23.5% in patients aged > 64 years), grade 1 to grade 3 loss of appetite (67.0% versus 73.8% versus 84.4%, respectively), grade 1 to grade 4 nausea and vomiting (77.5% versus 79.3% versus 85.5%, respectively), grade 1 to grade 4 diarrhea (40.6% versus 42.2% versus 50.6%, respectively), and raised creatinine levels grade 1 to grade 4 (3.4% versus 6.3% versus 7.8%, respectively) with the docetaxel regimen with increasing age. With paclitaxel, grade 3 to grade 4 mucositis (1.6% versus 5.9% versus 6.3%, respectively) and raised creatinine levels grade 1 to grade 4 (81.3% versus 4.2% versus 7.0%, respectively) significantly increased with age. Infection with neutropenia grade 1 to grade 4 overall was statistically significantly increased with age.

Conversely, there was a significantly higher incidence of grade 1 to grade 3 hot flushes (64.6% versus 59.1% versus 51.1%, *P *= 0.031) and grade 1 to grade 4 changes in liver enzymes (53.5% versus 50.3% versus 42.9%, *P *< 0.001) in younger patients versus older patients.

## Discussion

There is a growing awareness that age *per se *is a less important determinant of choice of therapy concepts and outcome in older cancer patients than physical status, functional status, and mental/emotional status. Indeed, it is becoming more and more evident that older, but otherwise healthy, patients with primary breast cancer can accrue the same benefits from standard chemotherapy as younger patients – especially when regarding the steady increase in life expectancy and quality of life in western societies. The clinical assumption that most older patients are too frail to receive standard chemotherapy – reflected in, for example, less use of adjuvant chemotherapy in older breast cancer patients [8-10] – is being challenged [11,12]. There is a paucity of published data from chemotherapy trials comparing older with younger cancer patients, however, which has hampered efforts to improve treatment strategies for this population. This lack of data arises from the common underrepresentation, if not routine exclusion, of older patients from many clinical trials [13-15]. Additionally, there are pharmacokinetic data demonstrating that both taxanes can be used in older patients without dose modifications but in the case of an impaired liver function neither paclitaxel nor docetaxel should be applied due to their high liver metabolization [16-18].

The present analysis constitutes the largest pooled analysis to date of the use of taxanes in older patients with primary breast cancer. The incidences of dose delays, dose reductions, treatment discontinuations, and hospitalizations all increased with age. With regard to taxane therapy, the combination dose-dense doxorubicin/docetaxel schedule was the most problematic overall, with a particularly high incidence of hospitalizations compared with other schedules. One cannot conclude, however, that nontaxane therapies are the preferred option. In a previous analysis of the data, the regimen of 5-fluorouracil, epirubicin on days 1 and 8 and cyclophosphamide on days 1 to 14 [19] was by far the most toxic regimen overall [20].

Amongst the taxane schedules, TAC was overall associated with the highest incidence of toxicity. In our analysis, paclitaxel had significantly less hematologic toxicity compared with docetaxel – which is consistent with data from a prospective adjuvant trial directly comparing those taxanes [21]. Moreover, in an adjuvant anthracycline/taxane sequential regimen, weekly paclitaxel shows the same efficacy as docetaxel [21]. This might therefore be the preferred taxane regimen, at least for older patients. The relatively low incidence of hematologic toxicity, with the exception of low-grade anemia, with the sequence dose-dense schedule is notable and may reflect the sequential dosing schedule and the obligatory use of granulocyte-colony stimulating factor therapy.

With regard to higher grade nonhematologic toxicity, mucositis, loss of appetite, infection with neutropenia, and fatigue were more common with increasing age. In patients treated with docetaxel, skin changes of grade 3 to grade 4 increased with age.

Analysis of the data by age shows that there was generally a higher incidence of dose delays/reductions, hospitalizations and therapy discontinuations, of hematologic toxicity, and of some nonhematologic toxicity such as loss of appetite and grade 3 to grade 4 fatigue and mucositis in 'older' versus younger patients. One might argue that such an age split in three different groups is artificial, that a higher age should be considered to constitute 'older' patients, and that simple discrimination by age alone does not account for other factors (such as physical status). Nevertheless, these data provide an important insight into the acceptable tolerability of chemotherapy in older patients with primary breast cancer and could be confirmed by data from patients with ovarian cancer [13].

The patient cohort for the four trials analyzed reflects clinical trial routine at that time and substantiates findings from other studies [22-24], demonstrating that – even if the trials had no upper age limit – older patients were generally included less frequently or not included. This may have changed since the data published by Muss and colleagues [25], which showed that the benefit from chemotherapy did not differ across age groups although treatment-related mortality was higher (1.5% versus 0.42%) in the group aged > 65 years.

While the present analysis has provided a wealth of important new data, it is important to acknowledge its limitations – namely, the *post hoc *nature of the analysis, a relative paucity of patients aged > 70 years, the probability that the patient population is not representative of older patients as a whole due to general exclusion of patients with comorbidities from clinical trials, and the lack of any exact prognostic score such as the Charlson score for enrolled older patients. Heterogeneous use of growth factors between the studies analyzed and their chemotherapy regimens may also have had an impact on some of the variables assessed.

In summary, our analysis indicates that – with pretreatment older assessment and appropriate supportive care such as granulocyte-colony stimulating factor therapy for neutropenia prophylaxis – older patients can be considered for taxane therapy and the maintenance of dose intensity should be feasible. Older patients generally have an increased susceptibility for myelotoxicity [26-28]; this has recently been acknowledged by the updated EORTC guidelines, which recommend general use of granulocyte-colony stimulating factor prophylaxis if the risk of FN is ≥ 20% and risk-adapted use in cases where the FN risk is between 10% and 20% [26]. Our findings are in accordance with other studies that have, for example, suggested docetaxel therapy as a viable treatment option in older cancer patients [29,30]. In particular, sequential therapy should be preferred among older patients. It has therefore become increasingly clear that (neo)adjuvant therapy concepts for older patients should consider – next to oncological needs – the patient's physical and functional status, and should not be determined merely on the basis of their age as in the ICE trial of the German Breast Group. Otherwise older patients will continue to be undertreated and will not benefit from the advances in medicine.

## Conclusion

The pooled analysis of a substantial cohort of older primary breast cancer patients demonstrates that taxane-containing (neo)adjuvant chemotherapy is feasible in older patients and that toxicity can be reduced by sequential therapy regimens and consequent use of prophylactic treatment.

## Abbreviations

FN: febrile neutropenia; TAC: docetaxel/doxorubicin/cyclophosphamide

## Competing interests

SL, WJ, DE, HE, VN, HS, and SK declare that they have no competing interests. GvM, MKi, and MKa received lecture honoraria from Sanofi-Aventis and Amgen. NH received lecture honoraria from Sanofi-Aventis and Amgen and consulting honoraria from Amgen.

## Authors' contributions

SL and VN had full access to all of the data in the study and take responsibility for the integrity and the accuracy of the data analysis. All of the authors had full responsibility in the design of the study, the collection of the data, the analysis and interpretation of the data, the decision to submit the manuscript for publication, and the writing of the manuscript.
